# Host-Guest Interactions of Plumbagin with β-Cyclodextrin, Dimethyl-β-Cyclodextrin and Hydroxypropyl-β-Cyclodextrin: Semi-Empirical Quantum Mechanical PM6 and PM7 Methods

**DOI:** 10.3390/scipharm86020020

**Published:** 2018-05-15

**Authors:** Ornin Srihakulung, Ryo Maezono, Pisanu Toochinda, Waree Kongprawechnon, Apichart Intarapanich, Luckhana Lawtrakul

**Affiliations:** 1 School of Bio-Chemical Engineering and Technology, Sirindhorn International Institute of Technology, Thammasat University, Pathum Thani 12120, Thailand; nam.ornin@gmail.com (O.S.); pisanu@siit.tu.ac.th (P.T.); 2 School of Information Science, Japan Advanced Institute of Science and Technology, Nomi, Ishikawa 923-1292, Japan; rmaezono@me.com; 3 School of Information, Computer and Communication Technology, Sirindhorn International Institute of Technology, Thammasat University, Pathum Thani 12000, Thailand; waree@siit.tu.ac.th; 4 National Electronics and Computer Technology Center, National Science and Technology Development Agency, Pathumthani 12120, Thailand; apichart.intarapanich@nectec.or.th

**Keywords:** plumbagin, cyclodextrin, inclusion complex, host–guest interaction

## Abstract

Molecular interactions of plumbagin inclusion complexes with β-cyclodextrin (BCD), dimethyl-β-cyclodextrin (MBCD), and hydroxypropyl-β-cyclodextrin (HPBCD) were investigated by semi-empirical, Parameterization Method 6 and 7 (PM6, and PM7) in the aqueous phase using polarizable continuum calculations. The results revealed two different binding modes of the plumbagin molecule inside the BCD cavity with a negative value of the complexation energy. In conformation-I, the hydroxyl phenolic group of plumbagin was placed in the BCD cavity near the narrow-side of the host molecule. In the other model, conformation-II, the methyl quinone group of plumbagin was placed in the cavity of BCD near the narrow-side of the host molecule. The higher the negative value of the complexation energy, the more favorable is the pathway of inclusion-complex formation.

## 1. Introduction

Molecular modeling techniques are currently widely used in chemistry and pharmacology to obtain insight into information at the molecular level of systems of interest. The computational results help explain the molecular interactions and suggest the mechanisms that govern the processes when experimental techniques are insufficient. The calculation models are able to predict and screen the results when varying the compounds or the system conditions prior to laboratory tests. Semi-empirical quantum mechanical calculations have been successful for descriptions in organic chemistry because some parameters are approximated or generalized to simplify the calculation or to yield a result based on experimental data. Modern semi-empirical quantum mechanical models such as Parameterization Method 6 and 7 (PM6 and PM7) are often used to explore the electronic structure dependent properties of large molecules, where ab initio electronic structure methods (without approximations) are too expensive [1]. The PM7 method improved the description of some properties such as the heats of formation or the height of the reaction barriers for reactions and included them into the description of the dispersion interaction and hydrogen bonding in the parameterization [2], which is suitable for a description of noncovalent interactions. In this work, the molecular interactions of a plumbagin inclusion complex with β-cyclodextrin (BCD), dimethyl-β-cyclodextrin (MBCD), and hydroxypropyl-β-cyclodextrin (HPBCD) were investigated. The inclusion complex formation takes place mainly by hydrophobic interactions—namely, van der Waals, hydrogen bonding, and dipole–dipole interactions—which are noncovalent interactions [3,4]. Therefore, the PM7 method should be appropriate to investigate the complexation mechanism of plumbagin with cyclodextrins. The results were also compared to the previous PM6 method.

β-cyclodextrin is a nontoxic cyclic oligosaccharide comprising seven α-D-glucoses [5]. The inner hydrophobic cavity of BCD consists of carbon and hydrogen atoms. The rims of the cavity comprise primary and secondary hydroxyl groups, giving it a hydrophilic property. The secondary hydroxyl groups are at the C2 and C3 positions of the cyclodextrin (CD), which is located on the wide-side of the truncated cone. The primary hydroxyl group is at the C6 position, which is located at the narrow-side of the truncated cone [3]. Methylated β-cyclodextrin and hydroxypropyl-β-cyclodextrin are BCD derivatives that are widely used in drug encapsulation because of their inclusion ability and high water solubility (>500 mg/mL) while the solubility in water of BCD is 18.5 mg/mL at 25 °C [6].

Plumbagin (5-hydroxy-2-methyl-1,4-naphthoquinone) is an organic compound extracted from the *Plumbago indica* root [7]. Plumbagin has anti-microbial, neuroprotective, and anti-carcinogenic properties and is generally used in Thai herbal medicines [8]. There are some reports using BCD and HPBCD to form a 1:1 inclusion complex with plumbagin to increase its solubility in water and reduce the cytotoxicity of the compound [9,10,11]. Unfortunately, no experimental papers report the use of MBCD as a host for encapsulation with plumbagin.

To understand the properties of the complex geometries and the encapsulation process, we used semi-empirical PM6 and PM7 methods to describe the complexation of plumbagin/BCD, plumbagin/MBCD, and plumbagin/HPBCD systems in water. The complex energy, molecular interactions, and insertion pathway of plumbagin/BCDs were examined.

## 2. Materials and Methods

### 2.1. Molecular Structure Construction

**Figure 1:**
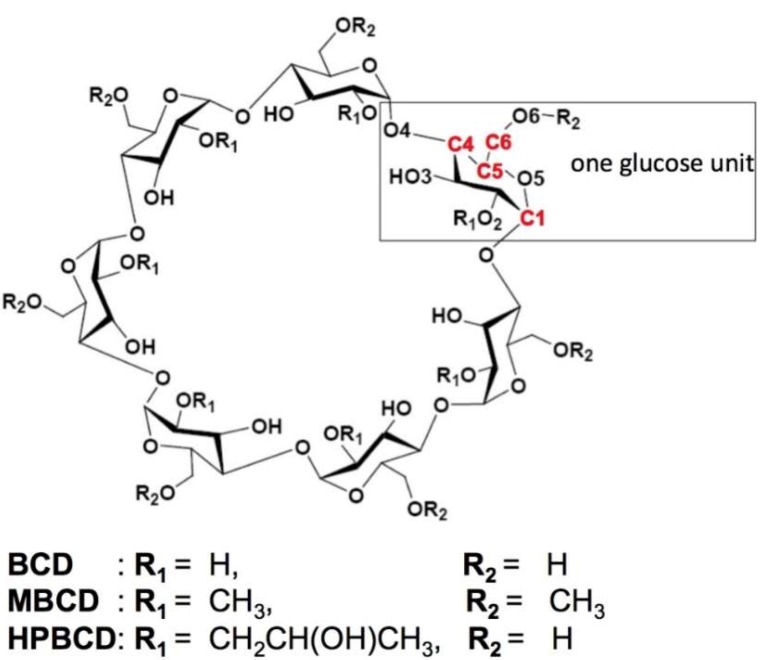
Schematic representations of glucose unit and atomic numbering of β-cyclodextrin (BCD), dimethyl-β-cyclodextrin (MBCD) and hydroxypropyl-β-cyclodextrin (HPBCD). All R_1_ and R_2_ are substituted by methyl groups on all of the glucose units in MBCD. In HPBCD, only the hydroxyl group at the R_1_ position of one glucose unit is substituted by a hydroxypropyl group.

**Figure 2:**
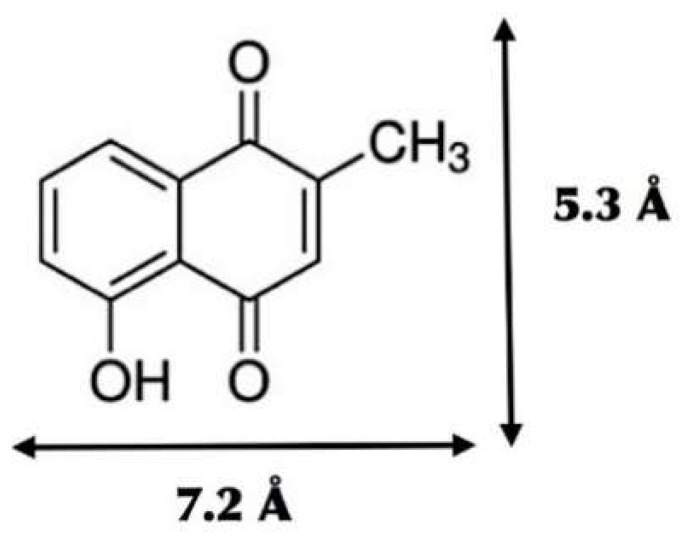
Chemical structure of plumbagin (C_11_H_8_O_3_) and the dimensions of its minimized molecular conformation.

All calculations were performed using GaussView 6.0 and Gaussian 16 software packages [12]. The crystal structure of plumbagin, BCD, MBCD, and HPBCD molecules were downloaded from Cambridge Crystallographic Data Centre [13] with the Cambridge Structural Database (CSD) Entry: PVVAQS01 [14], BCDEXD03 [15], BOYFOK04 [16], and KOYYUS [17], respectively (Figure 1 and Figure 2). Hydrogen atoms were added into the structures, and then fully optimized by the semi-empirical quantum mechanical PM6 and PM7 methods. The polarizable continuum model (PCM) [18] was used to model solvation effects for water as the solvent.

### 2.2. Molecular Docking Calculation

AutoDock 4.2.6 [19] with the Lamarckian genetic algorithm [20] was used to generate the possible conformations of plumbagin/BCDs inclusion complexes. AutoDock calculations were performed in four steps: (1) preparation of coordinate files using AutoDockTools, (2) pre-calculation of atomic affinities using AutoGrid, (3) docking of ligands using AutoDock, and (4) analysis of results using AutoDockTools. 

The first step was to prepare the guest (plumbagin) and host (BCDs) coordinate files to include the information needed by AutoGrid and AutoDock. The non-polar hydrogens were deleted and their charges were merged with the carbon atoms. The atom types were assigned, defining hydrogen bond acceptors and donors and aromatic and aliphatic carbon atoms. The rotatable bonds of the guests were defined while the hosts were kept fixed. AutoGrid was used to calculate the grid maps, one for each atom type present in the guest being docked. The systems were investigated in a three-dimensional volume divided into many small grid boxes with a grid spacing of 0.375 Å. The grid center of the boxes were set at the center of the host molecules. The box has x × y × z dimensions of 14.25 Å × 14.25 Å × 7.50 Å, 15.75 Å × 14.25 Å × 9.75 Å and 18.75 Å × 14.25 Å × 9.00 Å for BCD, MBCD, and HPBCD, respectively. AutoDock used the Lamarckian genetic algorithm to calculate the conformational states of a flexible guest, using the grid maps generated by AutoGrid to evaluate the guest–host interaction at each point in the docking simulation. One hundred docking calculations were performed on each guest–host complex. The results were clustered to identify similar conformations based on all-atom root mean square deviation within 2 Å. At the end of molecular docking calculations, AutoDockTools was used to perform a cluster analysis of the different docked conformations. The lowest energy representative docked conformation from molecular docking was selected for further full geometry optimization.

### 2.3. Complexation Energy Calculation

The selected docked conformation of plumbagin/BCD inclusion complexes was then fully geometry optimized by the PM6 and PM7 methods. All atoms were allowed to move freely in an aqueous environment. The most stable conformation of the plumbagin/BCD, plumbagin/MBCD, and plumbagin/HPBCD inclusion complexes were selected by considering the complexation energy (Δ*E*) as being the difference between the heat of formation of the complex and the heat of formation of the involved free molecules

(1) $$\Delta E=E_{PL/BCD}-\left(E_{PL}+E_{BCD}\right)$$

where *E_PL/BCD_*, *E_PL_*, and *E_BCD_* represent the heat of formation of the complex, isolated plumbagin molecule, and isolated BCD molecule, respectively.

## 3. Results and Discussion

### 3.1. Molecular Docking Calculation

**Figure 3:**
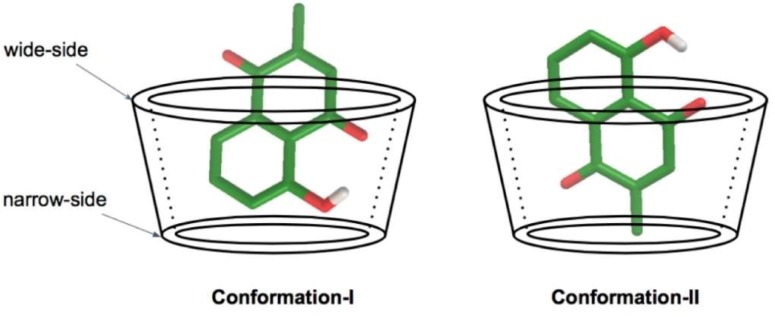
Schematic representation of two conformations of the inclusion complex. The secondary hydroxyl groups are at the C2 and C3 positions of the BCD, which are located on the wide-side of the truncated cone. The primary hydroxyl group is at the C6 position of the BCD, which is located at the narrow-side of the truncated cone.

Molecular docking was used to calculate the possibility of binding between a plumbagin molecule complex with each BCD by fixing the host structure and allowing the guest to be flexible in the specified grid box. The calculations indicated two possible conformations of the 1:1 guest:host ratio for all systems, as shown in Table 1 and Table 2. In conformation-I, the hydroxyl phenolic group of plumbagin was placed in the BCD cavity near the narrow-side of the host molecule. In the other model, conformation-II, the methyl quinone group of plumbagin was placed in the BCD cavity near the narrow-side of the host molecule, as illustrated in Figure 3.

Molecular docking results indicated that plumbagin/BCD and plumbagin/MBCD complexes in conformation-I were favorable while the plumbagin/HPBCD complex preferred conformation-II, using both of PM6 and PM7 minimized starting geometries. However, the rigidity of the host molecule in the docking calculations was not realistic. Therefore, the semi-empirical PM6 and PM7 methods in the aqueous phase using polarizable continuum calculations, were used to further investigate the molecular interactions of plumbagin with three different BCD hosts.

### 3.2. Complexation Energy Calculation

An inclusion complex of plumbagin with each of the BCD systems from docking calculations was generated. Both conformation-I and conformation-II, were then fully optimized by the PM6 and PM7 methods, which provide free motions for host and guest molecules in an aqueous environment. From PM6 and PM7 results (Table 3), the heat of formation of the minimized structure of the complex was always lower than that of the sum of the heat of formation of the isolated guest and host molecules indicating the formation of a favorable complex in all models. The complexation energy (ΔE), according to Equation (1), is also shown in Table 3. The more negative the value of the complexation energy, the more favorable the pathway of inclusion-complex formation. Table 3 shows the favorable formation of a 1:1 guest:host ratio of plumbagin with three type of BCD in both possible conformations.

The values of complexation energy (ΔE) from PM7 (−41.41 to −30.10 kcal/mol) were considerably lower than PM6 (−12.78 to −5.70 kcal/mol). The PM7 method includes the description of the dispersion interaction and hydrogen bonding [2] in the parameterization, and thus, should be suitable for the description of noncovalent interactions in plumbagin/BCD complexes. 

The difference in ΔE between the two conformations (conformation-I–conformation-II), is also presented in Table 3. The obtained results indicated that plumbagin/BCD inclusion complexes prefer conformation-I (BCD-I) in a water environment. For the inclusion complex formation of plumbagin with modified β-cyclodextrins (MBCD and HPBCD), both conformation-I and conformation-II are favorable.

Table 4 presents the distance of the intermolecular hydrogen bonds, which are found in PM6 and PM7 minimized inclusion complex structures. Three types of hydrogen bonds were established. The first one, which is often found in inclusion complex systems, is between an ether-like anomeric oxygen atom of the host molecule and a hydrogen atom of plumbagin’s hydroxyl group (O4_(host)_…H_(OH-PL)_). The second one, found only in plumbagin/BCD complexes, is from an oxygen atom of plumbagin’s carbonyl group and the hydrogen atom of the secondary hydroxyl group at O3 of BCD-I (O_(CO-PL)_…H_(O3H-BCD)_) and the primary hydroxyl at O6 of BCD-II (O_(CO-PL)_…H_(O6H-BCD)_). The third one, is found only in HPBCD-II, between an oxygen atom of plumbagin’s hydroxyl group and the hydrogen atom of the secondary hydroxyl at O2 of HPBCD (O_(OH-PL)_…H_(O2H-HPBCD)_). The molecular interactions of each host–guest system in an aqueous environment are further discussed below.

### 3.3. Plumbagin/β-cyclodextrin Inclusion Complex

**Figure 4:**
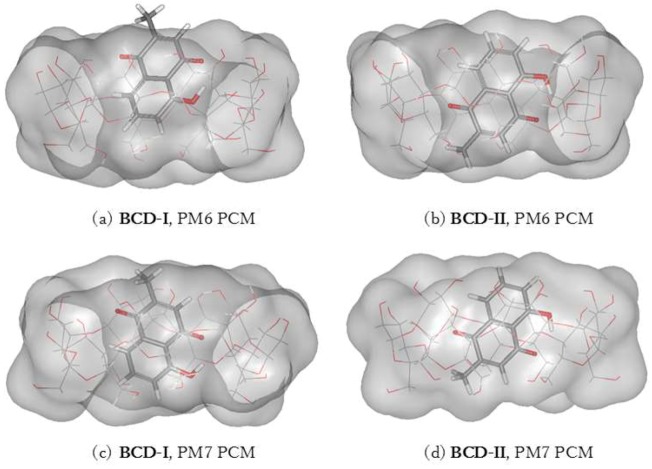
Energy-minimized structure of the 1:1 plumbagin/BCD complexes in an aqueous environment using polarizable continuum model (PCM). BCD is presented as a line model with a surface, with a probe radius of 1.4 Å. The plumbagin molecule is presented as a stick model. (**a**) plumbagin/BCD conformation-I obtained from PM6 method, (**b**) plumbagin/BCD conformation-II obtained from PM6 method, (**c**) plumbagin/BCD conformation-I obtained from PM7 method and (**d**) plumbagin/BCD conformation-II obtained from PM7 method.

**Figure 5:**
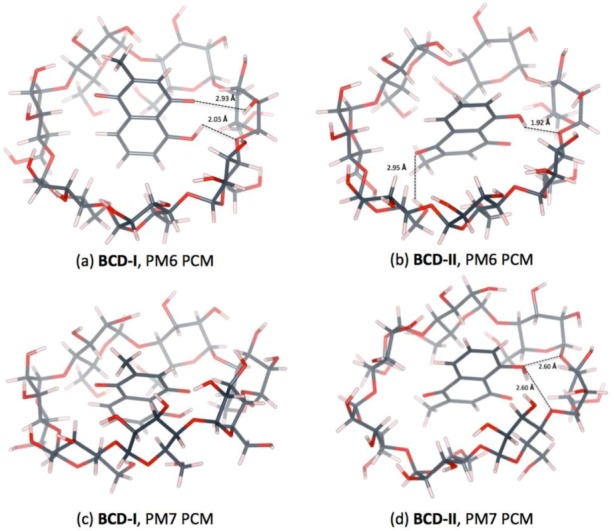
Hydrogen bonds in 1:1 plumbagin/BCD complexes. (**a**) plumbagin/BCD conformation-I obtained from PM6 method, (**b**) plumbagin/BCD conformation-II obtained from PM6 method, (**c**) plumbagin/BCD conformation-I obtained from PM7 method and (**d**) plumbagin/BCD conformation-II obtained from PM7 method.

Two conformations of a plumbagin/BCD inclusion complex can be formed in an aqueous environment, as shown in Figure 4. The intermolecular hydrogen bonds between plumbagin and BCD are depicted in Figure 5. BCD-I has a 0.03 and 2.37 kcal/mol lower complexation energy from the PM6 and PM7 methods, respectively, than BCD-II. The minimized plumbagin/BCD conformations obtained from the two methods were similar. However, in BCD-I from the PM7 calculation, the plumbagin molecule dipped deeper into the BCD cavity than the structure from the PM6 calculation. This occurred due to the hydrogen bond between the oxygen atom of plumbagin’s carbonyl group and the hydrogen atom of the secondary hydroxyl group at O3 of BCD-I (O_(CO-PL)_···H_(O3H-BCD)_), as shown in Figure 5a.

### 3.4. Plumbagin/Dimethyl-β-cyclodextrin Inclusion Complex

**Figure 6:**
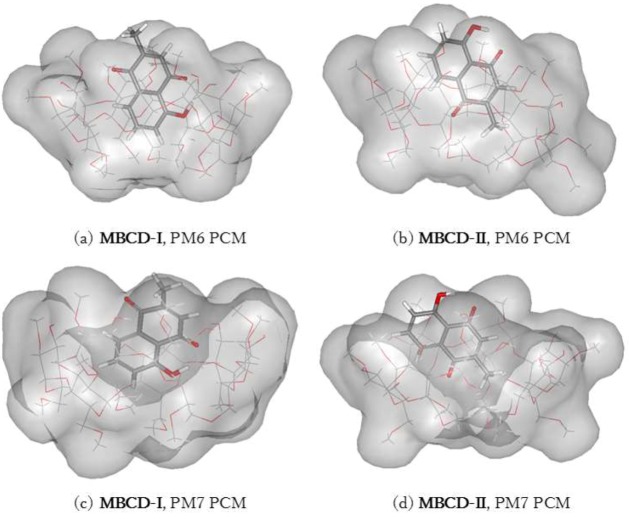
Energy-minimized structure of the 1:1 plumbagin/MBCD complexes in an aqueous environment using polarizable continuum model (PCM). MBCD is presented as a line model with a surface, with a probe radius of 1.4 Å. The plumbagin molecule is presented as a stick model. (**a**) plumbagin/MBCD conformation-I obtained from PM6 method, (**b**) plumbagin/MBCD conformation-II obtained from PM6 method, (**c**) plumbagin/MBCD conformation-I obtained from PM7 method and (**d**) plumbagin/MBCD conformation-II obtained from PM7 method.

**Figure 7:**
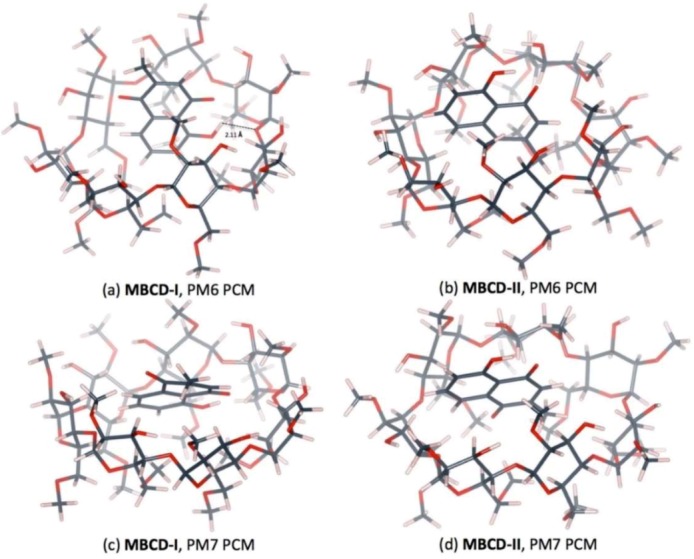
Hydrogen bonds in 1:1 plumbagin/MBCD complexes. (**a**) plumbagin/MBCD conformation-I obtained from PM6 method, (**b**) plumbagin/MBCD conformation-II obtained from PM6 method, (**c**) plumbagin/MBCD conformation-I obtained from PM7 method and (**d**) plumbagin/MBCD conformation-II obtained from PM7 method.

The plumbagin molecule is located near the wide-side of the MBCD molecule in all complex conformations, as shown in Figure 6 and Figure 7. These occurred due to the presence of methyl groups at the primary hydroxyl group of all glucose units (C6 position), condensing the cavity near the narrow-side of MBCD. In MBCD-I, without the steric hindrance from the guest molecule, all seven methoxy groups at the C6 position can be accommodated. After insertion of a plumbagin molecule in MBCD-II, two of the methoxy groups at the C6 position of MBCD move away from the cavity due to the presence of the methyl group of the plumbagin molecule, located at the narrow-side of MBCD, as seen in Figure 6b,d. According to the steric and electronic hindrances, plumbagin should enter into MBCD at the wide side to form the inclusion complexes MBCD-I and MBCD-II. The plumbagin/MBCD inclusion complex structures are very complicated. Using the same initial starting geometry, the energy-minimized conformations obtained from the PM6 and PM7 calculations were altered.

### 3.5. Plumbagin/Hydroxypropyl-β-cyclodextrin Inclusion Complex

**Figure 8:**
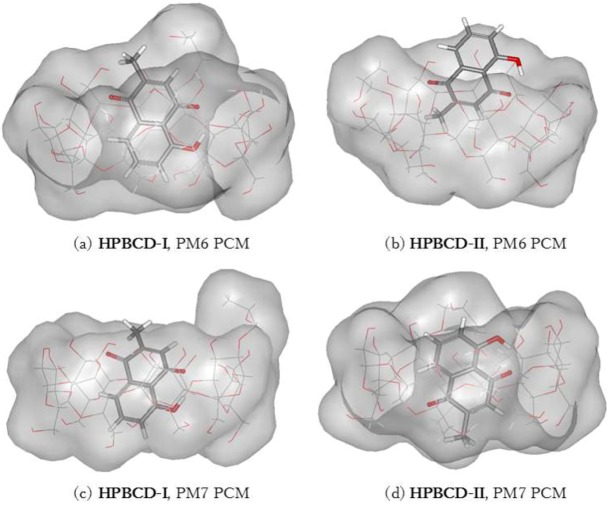
Energy-minimized structure of the 1:1 plumbagin/HPBCD complexes in an aqueous environment using polarizable continuum model (PCM). HPBCD is presented as a line model with a surface, with a probe radius of 1.4 Å. The plumbagin molecule is presented as a stick model. (**a**) plumbagin/HPBCD conformation-I obtained from PM6 method, (**b**) plumbagin/HPBCD conformation-II obtained from PM6 method, (**c**) plumbagin/HPBCD conformation-I obtained from PM7 method and (**d**) plumbagin/HPBCD conformation-II obtained from PM7 method.

**Figure 9:**
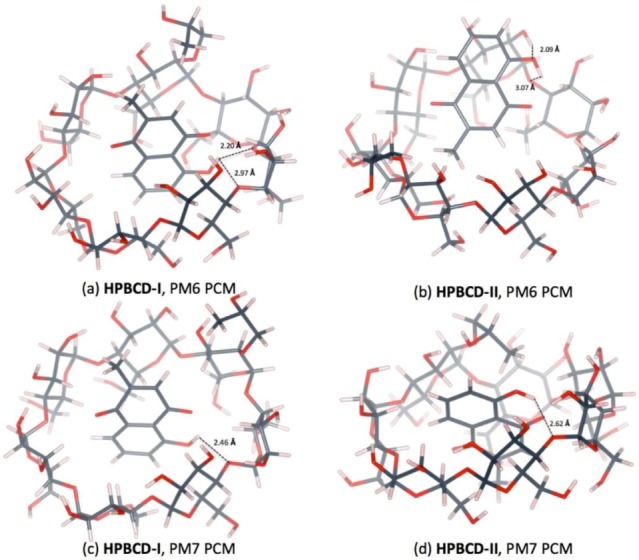
Hydrogen bonds in 1:1 plumbagin/HPBCD complexes. (**a**) plumbagin/HPBCD conformation-I obtained from PM6 method, (**b**) plumbagin/HPBCD conformation-II obtained from PM6 method, (**c**) plumbagin/HPBCD conformation-I obtained from PM7 method and (**d**) plumbagin/HPBCD conformation-II obtained from PM7 method.

The inclusion complex of plumbagin and HPBCD in conformation-I and conformation-II were stabilized in a water environment, as shown in Figure 8 and Figure 9. The presence of the hydroxypropyl group at the C2 position on a glucose unit in HPBCD enlarges the width of the wide-side. The energy-minimized structures of HPBCD-I from the PM6 and PM7 methods were similar. The hydroxypropyl group of HPBCD lined up in the parallel direction with the methyl group of the plumbagin molecule. The guest molecule is located inside the HPBCD’s cavity with an H-bond between the hydrogen atom of plumbagin’s hydroxy group and the ether-like anomeric oxygen atom of HPBCD.

PM6 and PM7 calculations yield different HPBCD-II energy-minimized structures in a water environment. In HPBCD-II, for the PM6 calculation, the guest molecule is located near the wide-side of HPBCD (Figure 8b), due to the H-bond which formed between the hydroxyl group of plumbagin and the secondary hydroxyl group at O2 of HPBCD (O_(OH-PL)_…H_(O2H-HPBCD)_), as mentioned in Table 4 and depicted in Figure 9b. Therefore, the guest molecule could not go deeper inside the HPBCD’s cavity, yielding HPBCD-I as the preferable complex with a lower complexation energy (3.38 kcal/mol) than HPBCD-II in the PM6 calculations. In the PM7 calculations, HPBCD-II was more favorable with a lower complexation energy (9.54 kcal/mol) than HPBCD-I. The methyl part of the hydroxypropyl group substituent falls into the HPBCD’s cavity and pushes the plumbagin molecule deeper inside the cavity due to the hydrophobic interaction.

## 4. Conclusions

The complexation energy values of each system obtained by the PM7 method are significantly lower than those obtained by the PM6 method. The obtained results agree with the experimental data for a 1:1 guest:host ratio of plumbagin with BCD and HPBCD inclusion complexes. We predict that by using MBCD to increase the solubility and reduce the cytotoxicity of the plumbagin compound, a 1:1 guest:host inclusion complex can be produced. Our results revealed two different binding modes of the plumbagin molecule inside the BCD cavity. In conformation-I, the hydroxyl phenolic group of plumbagin was placed in the BCD cavity near the narrow-side of the host molecule. In the other model, conformation-II, the methyl quinone group of plumbagin was placed in the cavity of BCD near the narrow-side of the host molecule. The intermolecular hydrogen bond, van der Waals, and hydrophobic interactions play an important role in complexation process of plumbagin with BCDs.

## Figures and Tables

**Table 1:** The lowest and the average values of free energy of binding (ΔG) of plumbagin/BCDs inclusion complexes and the number of conformations in a cluster (frequency) obtained from molecular docking calculations at 298.15 K. The starting geometry of the host and guest molecules were calculated by the PM6 method.

Guest/Host	Cluster	Conformation	Frequency (%)	ΔG (kcal/mol)	
				Lowest	Average
plumbagin/BCD	1	**I**	100	−6.21	−6.19
plumbagin/MBCD	1	**I**	85	−5.14	−5.13
	2	**II**	15	−5.03	−5.02
plumbagin/HPBCD	1	**II**	48	−5.76	−5.75
	2	**I**	2	−5.74	−5.73
	3	**I**	50	−5.72	−5.71

**Table 2:** The lowest and the average values of free energy of binding (ΔG) of plumbagin/BCDs inclusion complexes and the number of conformations in a cluster (frequency) obtained from molecular docking calculations at 298.15 K. The starting geometry of the host and guest molecules were calculated by the PM7 method.

Guest/Host	Cluster	Conformation	Frequency (%)	ΔG (kcal/mol)	
				Lowest	Average
plumbagin/BCD	1	**I**	61	−5.34	−5.26
	2	**I**	2	−5.24	5.23
	3	**II**	24	−5.22	−5.21
	4	**II**	13	−5.20	−5.18
plumbagin/MBCD	1	**I**	100	−5.12	−5.12
plumbagin/HPBCD	1	**II**	100	−5.89	−5.87

**Table 3:** Heat of formation energy (E) and complexation energy (ΔE) of the minimized inclusion complexes structures from PM6 and PM7 methods.

	PM6		PM7	
	E (kcal/mol)	∆E (kcal/mol)	E (kcal/mol)	∆E (kcal/mol)
*Isolated molecule*				
Plumbagin	−84.56		−87.09	
BCD	−1614.25		−1648.53	
MBCD	−1543.64		−1573.87	
HPBCD	−1659.94		−1701.90	
*Inclusion Complex*				
BCD-I	−1704.99	−6.18	−1768.09	−32.47
BCD-II	−1704.97	−6.15	−1765.72	−30.10
BCD-I–BCD-II		**−0.03**		**−2.37**
MBCD-I	−1636.23	−8.03	−1702.37	−41.41
MBCD-II	−1640.97	−12.78	−1699.12	−38.17
MBCD-I–MBCD-II		**4.75**		**−3.24**
HPBCD-I	−1753.57	−9.08	−1820.19	−31.21
HPBCD-II	−1750.20	−5.70	−1829.73	−40.75
HPBCD-I–HPBCD-II		**−3.38**		**9.54**

**Table 4:** Distance of hydrogen bonds between plumbagin (PL) and three different types of cyclodextrins (BCD, MBCD, and HPBCD) molecules, obtained from PM6 and PM7 minimized inclusion complex structures.

			Distance (Å)
PM6	BCD-I	O4_(BCD)_…H_(OH-PL)_	2.05
		O_(CO-PL)_…H_(O3H-BCD)_	2.93
PM6	BCD-II	O4_(BCD)_…H_(OH-PL)_	1.92
		O_(CO-PL)_…H_(O6H-BCD)_	2.95
PM6	MBCD-I	O4_(MBCD)_…H_(OH-PL)_	2.11
PM6	HPBCD-I	O4_(HPBCD)n_…H_(OH-PL)_	2.20
		O4_(HPBCD)n+1_…H_(OH-PL)_	2.97
PM6	HPBCD-II	O4_(HPBCD)_…H_(OH-PL)_	3.07
		O_(OH-PL)_…H_(O2H-HPBCD)_	2.09
PM7	BCD-II	O4_(BCD)n_…H_(OH-PL)_	2.60
		O4_(BCD)n+1_…H_(OH-PL)_	2.60
PM7	HPBCD-I	O4_(HPBCD_ …H_(OH-PL)_	2.46
PM7	HPBCD-II	O4_(HPBCD)_…H_(OH-PL)_	2.62
